# Attacins: A Promising Class of Insect Antimicrobial Peptides

**DOI:** 10.3390/antibiotics10020212

**Published:** 2021-02-20

**Authors:** Francesco Buonocore, Anna Maria Fausto, Giulia Della Pelle, Tomislav Roncevic, Marco Gerdol, Simona Picchietti

**Affiliations:** 1 Department for Innovation in Biological, Agro-food and Forest systems, University of Tuscia, Largo dell’Università snc, 05100 Viterbo, VT, Italy; fausto@unitus.it (A.M.F.); Giulia.della.pelle@ijs.si (G.D.P.); picchietti@unitus.it (S.P.); 2 Department of Biology, Faculty of Science, University of Split, Rudera Boskovica 33, 21000 Split, Croatia; troncevic@pmfst.hr; 3 Department of Life Sciences, University of Trieste, Via Giorgieri 5, 34127 Trieste, TS, Italy; marco.gerdol@gmail.com

**Keywords:** insect AMPs, attacins, antimicrobial activity, pharmacological applications

## Abstract

Insects produce a large repertoire of antimicrobial peptides (AMPs) as the first line of defense against bacteria, viruses, fungi or parasites. These peptides are produced from a large precursor that contains a signal domain, which is cleaved in vivo to produce the mature protein with antimicrobial activity. At present, AMPs from insects include several families which can be classified as cecropins, ponericins, defensins, lebocins, drosocin, Metchnikowin, gloverins, diptericins and attacins according to their structure and/or function. This short review is focused on attacins, a class of glycine-rich peptides/proteins that have been first discovered in the cecropia moth (*Hyalophora cecropia*). They are a rather heterogeneous group of immunity-related proteins that exhibit an antimicrobial effect mainly against Gram-negative bacteria. Here, we discuss different attacin and attacin-like AMPs that have been discovered so far and analyze their structure and phylogeny. Special focus is given to the physiological importance and mechanism of action of attacins against microbial pathogens together with their potential pharmacological applications, emphasizing their roles as antimicrobials.

## 1. Introduction

Antimicrobial peptides (AMPs) are important effector molecules of innate immunity [1] that can act against bacteria, viruses, fungi or parasites. The mechanism of action of these peptides, which often involves non-specific membrane interactions, prevents the development of long-lasting resistance by pathogens [2]. Although ubiquitous in nature, AMPs of animal origin can be found, from invertebrates to humans, in outer and inner epithelia [3], in phagocytic cells [4,5] and body fluids [6], providing a first line of host defense to eliminate invading pathogens [7,8] and boost immune responses [9].

So far, more than 3000 AMPs have been isolated from prokaryotes and eukaryotes, with an ever-growing fraction predicted via bioinformatic approaches [10,11] and genome mining [12,13,14]. A large fraction of protein sequences deposited in UniProt (i.e., 180,000,000), in addition to those reviewed and confirmed, are derived from bioinformatic prediction. Therefore, several undiscovered AMPs are likely to be included among the unreviewed sequences found in TrEMBL (Translated EMBL Nucleotide Sequence Data Library).

Since AMPs most likely derive from multiple independent evolutionary events, these molecules display an astounding diversity among taxa [2,15,16], but, at the same time, they share some common physicochemical properties that allow their classification into broad categories.

AMPs are produced from relatively short proteins usually containing 50–300 amino acids with a MW between 5 and 30 kDa. These precursors are cleaved by proteases to produce biologically active (mature) peptides that are mostly composed of 10–30 residues. Furthermore, amino acids are often arranged in an amphipathic manner, allowing for a certain clusterization of polar/charged and hydrophobic residues within the peptide sequence, which results in spatial separation in the active peptide [17,18,19]. They are usually composed of a large percentage of basic residues, often higher than 30% [20,21], which confers a positive net charge to most of the known peptides [22]. The cationic nature of AMPs allows their interaction with the cytoplasmic membranes of both Gram-negative and Gram-positive bacteria which are rich in phospholipids characterized by a negatively charged head group [23]. Moreover, they can also target the fungi cell wall and destroy the virus envelope or the parasitic protozoa membrane [24].

Therefore, the mechanism of action against these pathogens often involves membrane interaction [21,25], followed by the reaching of a critical peptide concentration [21,26,27,28] and subsequent membrane lysis. The latter can occur by toroidal pore, carpet like [29,30,31,32], and barrel stave [33] modes of action, with the barrel stave being recently called into question. However, numerous exceptions to these rules of thumb exist, and for more details we refer the reader to the following reviews [34,35,36].

A unifying theory about the mode of action of AMPs based on the thermodynamic interaction between the molecule and the lipid double layer, the so-called “graded/all or nothing” model, has been proposed by Almeida’s group [21,37]. Moreover, recent work focused on the molecular dynamics of the interaction between AMPs and phase separated model membranes [38], which demonstrated that the peptides showed a clear preference towards liquid-disordered phases. On the other hand, a certain number of AMPs show a different behavior and act in a non-lytic manner, preferring intracellular targets [39,40], operating as membrane transporter blockers [41], inhibiting cell division [42] and interacting with nucleic acids [43,44,45] and/or with the protein biosynthesis process [42].

## 2. Insects Antimicrobial Peptides: A Brief Insight

Having colonized nearly every ecological niche, insects can be considered the most successful animal taxon on Earth [46,47]. Their large AMP repertoire may be reasonably related to various environmental threats faced by each species during evolution [48]. Furthermore, their innate immune system is strictly interconnected with their endosymbionts, most often bacteria [49,50], which still retain immunoregulatory activity despite having lost most of their biosynthetic pathways. These endosymbionts reside in specialized cells called bacteriocytes [51] and the most represented genera are *Wolbachia*, *Spiroplasma* and *Wigglesworthia* [52]. The relation between the expression of insect AMPs and the control of these endosymbionts has only been recently clarified in detail [49,53].

In insects, AMPs are usually produced locally at various surface epithelia or secreted systemically by hemocytes and fat bodies into the hemolymph [48,54]. The release of the peptides in the hemolymph is triggered by the recognition of the pathogen by pattern-recognition receptors [55]. Moreover, in *Drosophila* it has been demonstrated that the ofteninterchangeable Toll and IMD (immune deficiency)-signaling pathways [56,57] regulate, respectively, Gram-negative and Gram-positive responses with the so-called “immune loitering” effect [55,58]. However, the relationship between the immune response mediated by AMPs and “acquired immunity” towards specific pathogens in insects likely relies on more complex regulatory mechanisms [49].

Although AMPs have been known since 1939 [15] (i.e., the first isolated peptides were gramicidins from the soil bacteria *Aneurinibacillus migulanus*), it was not until 1981 that the first insect antimicrobial peptide, named cecropin, was identified from the hemolymph of the *Hyalophora cecropia* pupae [59,60]. Several AMPs that can be classified according to their structure or function in different families (such as cecropins, ponericins, defensins, lebocins, drosocin, Metchnikowin, gloverins, diptericins and attacins) are presently known in insects.

Cecropins are cationic peptides of 31–39 amino acid residues that have been identified in lepidopteran, dipteran and coleopteran insects [61]. They show, as do most AMPs, a random coil structure in aqueous solution but convert to an α-helical linear structure in a hydrophobic environment [20,43]. Cecropins are active against fungi, Gram-negative and Gram-positive bacteria [61]. Some cecropin-like peptides have been also identified and assigned different names such as sarcotoxin, papiliocin, stomoxyn, hinnavin, bacteridicin, hyphancin and enbocin [62].

Ponericins are isolated from the venom of the predatory ant *Pachycondyla goeldii*. They are classified, based on their primary structure, into ponericins G, W and L [63], which show high sequence homology with other insect AMPs: cecropin, gaegurins/melittin and dermaseptins, respectively. Ponericins exhibit insecticidal and hemolytic activity against cricket larvae [62].

Insect defensins are small inducible cationic peptides with six cysteines [61,64]. Their structure, stabilized by three disulfide bridges, is composed of an N-terminal loop followed by an antiparallel β-sheet. AMPs belonging to the defensins family have been identified in different insect orders including Diptera, Hymenoptera, Coleoptera, Trichoptera, Hemiptera and Odonata [65]. Most defensins are active against Gram-positive bacteria, including some human pathogenic bacteria such as *Staphylococcus aureus*, and a few are also active against Gram-negative bacteria and fungi [61].

Other insect AMPs, including lebocins, drosocin, Metchnikowin, gloverins and diptericins, show a high percentage of specific amino acid residues, such as proline and/or glycine. Lebocins were first identified in the hemolymph of *Bombix mori* after immunization with *Escherichia coli*. They consist of 32 amino acids with a high fraction of proline residues and are prone to O-glycosylation. Lebocin precursors have been isolated in different lepidopteran species and they demonstrate broad biological activity against Gram-negative and Gram-positive bacteria, as well as some fungi [61]). Drosocin and Metchnikowin are produced by *Drosophila melanogaster* [66,67]. The former is a proline-rich peptide of 19 amino acids and is O-glycosylated, which is crucial to maximize its biological activity. The latter is a 26-residue proline-rich peptide which exhibits antibacterial activity against Gram-positive bacteria and also has antifungal properties. Gloverins are glycine-rich antimicrobial peptides exclusively present in Lepidopteran insects. The first gloverin peptide was identified in the hemolynph of *Hyposphora gloveri* pupae [68]. Gloverins shows a broad spectrum of antimicrobial activity, with some members of this family only being active against Gram-positive bacteria and others only against Gram-negative bacteria or viruses [61]. Finally, diptercins belong to a family of glycine-rich AMPs found in *Dipteran* hemolymph with an *Mr* of about 8000. The most important member of the family is diptericin A, which is mainly active against Gram-negative bacteria [62]. We decided to focus this Mini-review on attacins, providing a description of their first isolation and highlighting their known antimicrobial activities. Due to their higher molecular mass compared to most other AMPs, attacins need to be produced as recombinant molecules to test their biological activity. The widespread occurrence of attacins in several orders of insects highlights the evolutionary success of this class of AMPs and justifies the interest in their potential pharmacological applications.

## 3. Attacins

### 3.1. First Identification and Main Structural Features

The first paper reporting the identification of a new insect AMP family named attacin, from the giant silk moth *Hyalophora cecropia*, is dated 1983 [69]. Different isoforms with molecular masses of 20 to 23 KDa and isoelectric points (pI) ranging from 5.7 to 8.3 were purified from the hemolymph after the immunization of the pupae with the bacterium *Enterobacter cloacae* β12. The six isolated AMPs were classified in two groups: a basic group formed by attacins A to D, and an acidic one comprising attacins E and F.

Attacin F, which was demonstrated to represent a proteolytically derived product of attacin E [70], was produced as a recombinant protein by using the baculovirus expression system utilizing *Autographa californica* nuclear polyhedrosis virus (AcNPV) in *Spodoptera frugiperda* cells [71]. Interestingly, the two acidic attacin peptides and three out of four basic attacin peptides showed identical N-terminal sequences. This was confirmed by the isolation of only two cDNA clones for all attacins, one for each of the main isoforms [72]. The mature proteins exhibited a high degree of homology (76% on the nucleotide level and 79% on the amino acid level), while the homology dropped to lower levels in the 3′UTR region.

**Figure 1:**
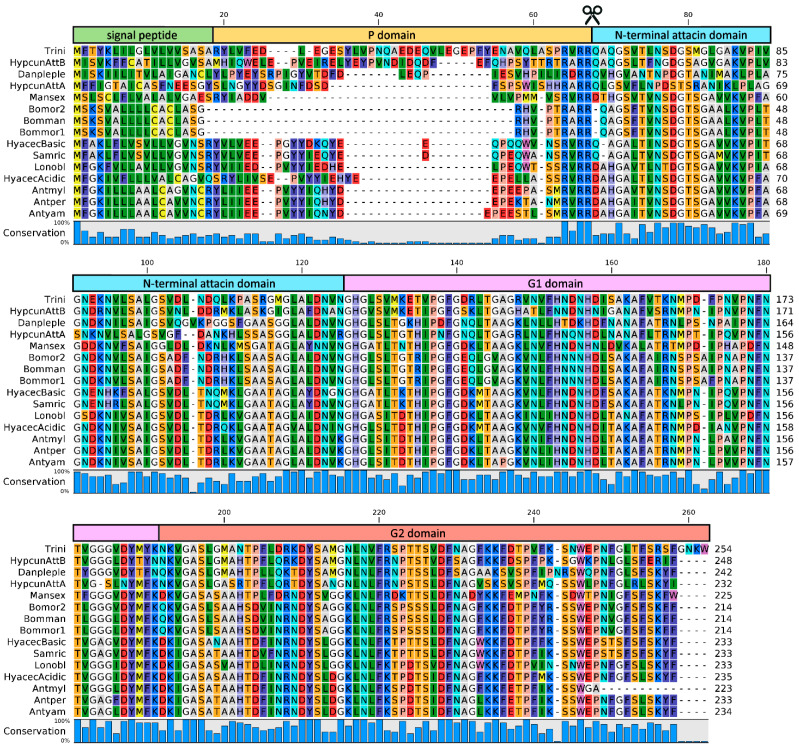
Alignment of attacin amino acid sequences from Lepidoptera obtained by Clustal ω. The predicted signal peptide and the different domains are highlighted above the sequences, together with the RXXR motif (indicated as cut symbol). Accession numbers: *Trichoplusia ni* U46130; *Hyphantria cunea* Attacin B ABL63641; *Danaus plexippus plexippus* OWR54405; *Hyphantria cunea* Attacin A AAD09288; *Manduca sexta* AAY82587; *Bombyx mori* 2 Q26431; *Bombyx mandarina* XP_028041931; *Bombyx mori* 1 ADB08384; *Hyalophora cecropia* Basic CAA44179; *Samia ricini* BAB69462; *Lonomia obliqua* Q5MGE6; *Hyalophora cecropia* Acidic CAA40886; *Antheraea mylitta* ABG72695; *Antheraea pernyi* ACB45562; *Antheraea yamamai* AFS30776. Sequence conservation is shown as a histogram below the multiple sequence alignment; Att = Attacin.

The expression of both attacin genes from *Hyalophora cecropia* could be upregulated by the stimulation of the pupae with phorbol 12-myristate 13-acetate (PMA), lipopolysaccharide (LPS from *Escherichia coli* D21) and bacteria (*Enterobacter cloacae* β12) [73]. The gene coding for the acidic attacin isoform displayed a faster response to the bacterial infection and it was the only one to show modulation after injury [73]. As most AMPs, attacins are produced as pre-pro-proteins. The pro-protein is formed by a pro-peptide (the P domain) and a mature peptide which consists of an N-terminal attacin domain and two glycine-rich (G1 and G2) domains (see Figure 1) [72,74]. The mature peptide is obtained through proteolytic cleavage by a furin-like enzyme, which recognizes a conserved RXXR motif found in the N-terminal region of the pro-protein; this sequence needs to be removed for efficient secretion of attacins from fat body cells [71].

Attacins adopt a random coil secondary structure in an aqueous buffer and, unlike many known AMPs, they do not show any transition to helical conformation in an anisotropic environment [73]. This is mostly due to the glycine-rich nature of these peptides, together with the lack of cysteine bridges that could stabilize their structure. Their complete mature region is approximately 190 amino acids in length, with net charges ranging from −3 to +10 (see Table 1). Since attacins are relatively large proteins, their net charge may not be detrimental for antimicrobial activity, as is often the case for shorter AMPs. Similarly, other important physicochemical properties correlated with the potency of peptides, such as relative hydrophobicity and amphipathicity, do not necessarily provide reliable information linked with their antimicrobial activity that most likely rely on a different mode of action. Indeed, the activity of attacins is probably linked with certain sections of the sequence with particular chemo-physical properties (e.g., positive charge, abundance of hydrophobic residues), that could be responsible for membrane interaction and subsequent antimicrobial activity.

### 3.2. Phylogenetic Considerations

**Figure 2:**
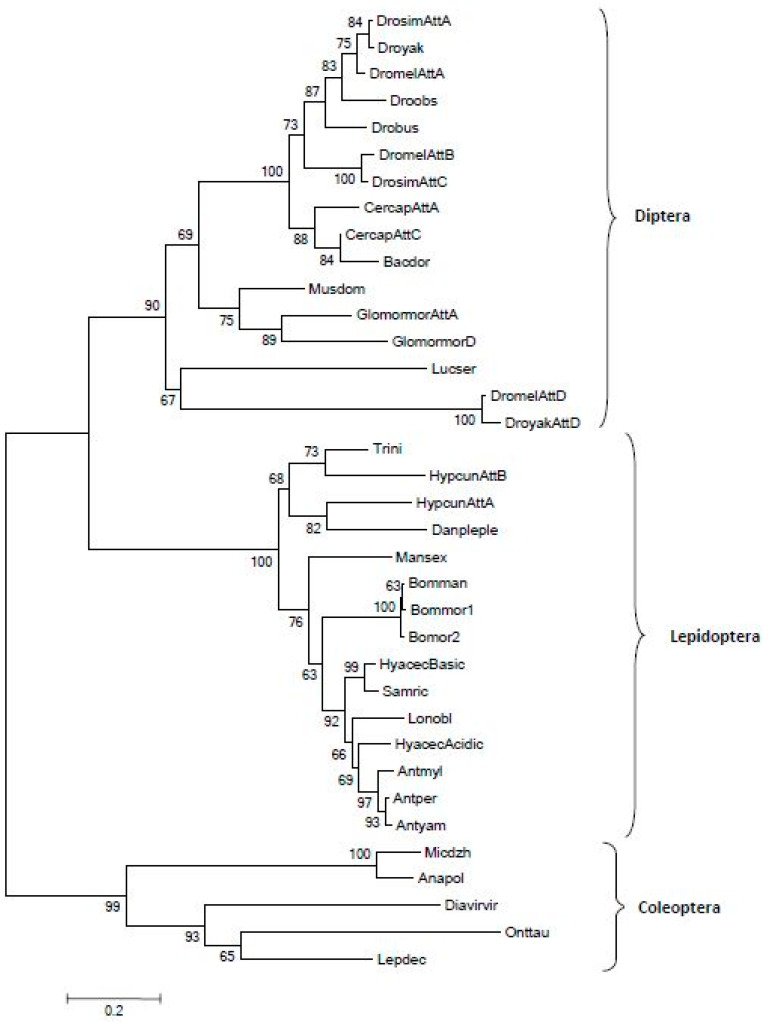
Phylogenetic tree of attacins from different insect orders obtained using the MEGA 6.0 program. The percentage of replicate trees in which the associated taxa clustered together in the bootstrap test (10.000 replicates) is shown next to the branches. The number 0.2 represents the genetic distance. Accession numbers: *Drosophila simulans* Attacin A EDX07123; *Drosophila yakuba* EDW91058; *Drosophila melanogaster* Attacin A Z46893; *Drosophila obscura* XP_022225780; *Drosophila busckii* ALC41796; *Drosophila melanogaster* Attacin B AAL23652; *Drosophila simulans* Attacin C AAP69835; *Ceratitis capitata* Attacin A XP004517761; *Ceratitis capitata* Attacin C XP004517762; *Bactrocera dorsalis* KJ598077; *Musca domestica* AAU08203; *Glossina morsitans morsitans* Attacin A AAL34113; *Glossina morsitans morsitans* Attacin D CAP78962; *Lucilia sericata* HM243534; *Drosophila melanogaster* Attacin D AAF55446; *Drosophila yakuba* Attacin D EDW95807; *Microdera dzhungarica* AHH34162; *Anatolica polita* APX53001; *Diabrotica virgifera virgifera* AHB11276; *Onthophagus taurus* XP_022912795; *Leptinotarsa decemlineata* XP_023024624. For the Lepidoptera accession numbers, see Figure 1. Att = Attacin.

After the first identification in *Hyalophora cecropia*, various attacin-like peptides have been discovered in many lepidopteran, dipteran and coleopteran species. Figure 2 shows a phylogenetic tree obtained from a multiple sequence alignment of attacin amino acid sequences. The peptides are divided in three different clades corresponding to the respective insect order they were isolated from. In the Diptera clade, multiple tandemly duplicated genes are usually present. In *Drosophila* for example, attacins A and B are placed close to each other in the same chromosome, whereas the phylogenetically distant attacin D is located on a different chromosome [74]. The comparison between attacins and other classes of AMPs found in insects evidenced that they share some similarities with the protein domains of sarcotoxin II and diptericin [73,74]. This homology is mainly evident within exon 2 and exon 3 of the attacins, which encode the G1 and G2 domains, although diptericin contains only a single glycine-rich domain. Since these two exons also show a significant pairwise sequence homology, it has been proposed that the three different AMP classes may have evolved from a single ancestral exon [73]. The C-terminal attacin domain (PFAM number PF03769) has been found in most of the Holometabola genomes studied so far, and in the orders Orthoptera, Isoptera and Hemiptera (where the encoded peptide is named prolixicin) [65]. Finally, attacin-like sequences have been very recently identified in the stick insect *Peruphasma schultei* (Pseudophasmatidae) after an infection with a microbial cocktail [75].

### 3.3. Antimicrobial Activity of Attacins and Therapeutical Applications

Generally, the available data show that attacins are active against both Gram-negative bacteria and Gram-positive bacteria, with certain selectivity towards Gram-negative strains [59,60]. Due to their size, attacins need to be produced as recombinant proteins prior to antimicrobial characterization (see the cut symbol in the multiple sequence alignment displayed in Figure 1).

The initial discovery of attacins in the giant silk moth immediately led to extensive investigations, allowing significant data collection about their antimicrobial activity within one year [69]. This was characterized by the sub-micromolar activity of attacin B and E against *Acinetobacter calcoaceticus*, *Pseudomonas maltophilia* and *Escherichia coli*, all representatives of Gram-negative bacteria (see Table 1).

Carlsson et al. [76] confirmed this activity against *Escherichia coli*, linking it to the alteration of the outer bacterial membrane through specific inhibition of the synthesis of several membrane proteins. More importantly, the same group showed that attacin was active against *Proetus mirabilis* polymyxin-resistant cells at a concentration 100-fold lower than that of polymyxin [76]. Given the fact that polymyxin is being successfully used to treat Gram-negative infections [77], this undoubtedly sparked the interest in future attacin-related research. These studies also showed that attacins trigger the expression of several stress proteins in the bacteria upon interaction with their receptor (i.e., bacterial lipopolysaccharide ); therefore, growth inhibition and stress induction do not require the entry of attacins into the cells [76].

Attacin A from *Drosophila melanogaster*, expressed as a recombinant protein in *E. coli*, exhibited antimicrobial activity against the Gram-negative bacteria *E. coli* DH5α and, at a lesser extent, *Stenotrophomonas maltophilia* (Table 1). Moreover, the peptide did not show any hemolytic activity on porcine red blood cells, which is especially important in terms of possible therapeutic applications, as it demonstrated selectivity against bacterial cell membranes [78].

Two attacin genes named attacin A and attacin B have been identified in the fall webworm *Hyphantria cunea* [79]. Attacin A and attacin B are leucine-rich and glycine-rich peptides, respectively, which displayed an opposite trend of expression in response to infection of larvae with bacteria, fungi and viruses. While the former was poorly induced, the latter underwent strong upregulation. Recombinant attacin A showed no antibacterial and antifungal activity, while recombinant attacin B was active against the Gram-negative bacteria *E. coli* and *Citrobacter freundii* and the fungus *Candida albicans* (Table 1).

Another attacin gene named seattacin was isolated from the beet armyworm (*Spodoptera exigua*). It was strongly upregulated 12 h after the stimulation of larvae with UV-killed bacteria *E. coli* and *Bacillus subtilis* (Gram-positive). The mature peptide was expressed in *E. coli*, and this recombinant molecule showed antimicrobial activity against *E. coli* DH5α, *Pseudomonas cichorii* (Gram-negative) and two Gram-positive species, *B. subtilis* and *Listeria monocytogenes* [80].

The expression of attacin A1, found in the fat body tissue of the tsetse fly *Glossina morsitans morsitans*, the vector of African trypanosome, was found to be highly induced after an infection with the protozoan agent. The purified attacin A1 recombinant peptide, expressed in the *Drosophila* S2 cells, showed strong antimicrobial activity against *E. coli* K12 and in vitro inhibitory effects against the mammalian bloodstream form and the insect stage of *Trypanosoma brucei* [81]. In particular, this last aspect has been considered of high pharmacological potential.

Another attacin-like peptide was identified in the black soldier fly *Hermetia illucens* [82]. The putative mature molecule, showing 50% homology with the attacin B from the oriental fruit fly *Bactrocera dorsalis* [83], was produced as recombinant protein in the inclusion bodies of *E. coli*. This attacin displayed antibacterial activity against *E. coli* and, more interestingly, against the *Staphylococcus aureus* KCCM 40881, a methicillin-resistant *Staphylococcus aureus* (MRSA). This is extremely important in terms of possible pharmacological applications.

Attacins have also been determined as one of the components of larval (*Lucilia sericata*) secretions that evidenced a positive effect in the chronic wound treatment of 30 patients involved in a clinical trial [84]. In recent years, the use of live maggots in biosurgery has been taken into consideration due to the possibility of disinfecting and cleaning infected injuries [84]. In the study by Wollina and colleagues [84], the secretion from the common green bottle fly showed a positive effect on tissue oxygenation, edema and the development of red granulation tissue. It was demonstrated that this effect was not only due to embryonic growth stimulating substances [84], but also to the presence of attacin peptides with antimicrobial effects on both Gram-negative and Gram-positive bacteria [85]. No in vivo data are presently available concerning attacin toxicity in mammal models. However, very recently the antimicrobial activity of a sarcotoxin from *Lucilia sericata*, which is structurally similar to attacins, was investigated against multiple drug resistant (MDR) pathogens [86]. A peptide concentration of 4 mg/l inhibited 90% of the tested MDR isolates. The sarcotoxin showed neither hemolytic nor cytotoxic effects on human cells. Moreover, in vivo tolerability studies in mice revealed that the peptide is not toxic and has better tolerance compared to colistin, a drug that has already been approved for clinical use [86].

## 4. Conclusions

In conclusion, attacins are certainly an interesting and still under-studied class of insect AMPs that deserve further investigations aimed at elucidating their potential pharmacological applications. Although some preliminary results on the antimicrobial activity of attacins against both methicillin-resistant bacteria and infective protozoan agents are available, several limitations need to be overcome. For instance, the clinical importance of AMPs resides in the fact that is quite challenging for microbes to develop permanent resistance, as this would imply preserving cell membrane functionality and integrity while simultaneously avoiding the action of AMPs. However, recent studies have shed new light on the mechanisms by which bacteria may develop resistance under selective pressure [23,87], providing a solid ground for the possible applications of AMPs.

Another key aspect that limits the possibilities of AMPs in a clinical context is their toxicity towards host cells which is often significant, as in the case of the bee venom peptide melittin, which is extremely potent towards bacteria, but at the same time toxic towards their host cells at similar concentrations which are needed to inhibit or kill bacteria [88]. This kind of data is still lacking for the majority of known attacin sequences and certainly deserves appropriate investigation in the future.

Finally, for a possible therapeutic application, it is fundamental to study the peptide stability in physiological conditions (i.e., the presence of salt, serum and proteases), as they are often quickly degraded. To overcome these setbacks concerning low stability and high toxicity, peptides can be synthetically modified in several ways. Some protective modifications may include cyclization (linking the C and N-terminus), the introduction of *D* isomers and/or the incorporation of non-proteinogenic amino acids in the peptide sequence [22].

However, these approaches are relatively complicated and, more importantly, they can increase the final production cost of AMPs, which is therefore usually higher than that of already available antibiotics. Further research is in progress to improve the low success rate of AMPs clinical applications compared to the high number of new peptides identified each year [24].

## Figures and Tables

**Table 1:** Attacins mature sequences (N-terminal, G1 and G2 domains) and antimicrobial activity.

Name	Sequence	Charge	Pathogen	Antimicrobial Activity
Attacin B from *Hyalophora cecropia*	QAGALTINSDGTSGAV-VKVPITGNENHKFSALGSVDLT-NQMKL	+3	*E. coli* D21	0.6 µM (inhibition zone assay)
	GAATAGLAYDNGNGHGATLT KTHIPGFGDKMTAAGKVNLFHN		*E. coli* D22	0.06 µM
	DNHDFSAKAFATKNMP-NIPQVPNFNTVGAGVDYMFKDKIG		*P. maltophilia* Pm1	4 µM
	ASANAAHTDFINRNDYS-LGGKLNLFKTPTTSLDFNAGWKKF		*A. calcoaceticus* Ac11	0.4 µM
	DTPFFKSSWEPSTSFSFSKYF			
Attacin E from *Hyalophora cecropia*	DAHGALTLNSDGTSGAVVKVPFAGNDKNIVSAIGSVDLT-DRQKL	−3	*E. coli* D21	2 µM (inhibition zone assay)
	GAATAGVALDNINGHGLSLTDT HIPGFGDKMTAAGKVNVFHNDNHD		*E. coli* D22	0.3 µM
	ITAKAFATRNMPDIANVPN FNTVGGGIDYMFKDKIG		*P. maltophilia* Pm1	15 µM
	ASASAAHTDFINRNDYSLDGKLNLFKTPDTSIDFNAGFKKFDTPFMKSS		*A. calcoaceticus* Ac11	1 µM
	WEPNFGFSLSKYF			
Attacin A from *Drosophila melanogaster*	QVLGGSLTSNPAGGADARLDLT-KGIGNPNHNVVGQVFAAGNTQSG	+3	*E. coli* DH5α	N.D.
	PVTTGGTLAYNNAGHGASLTKT HTPGVKDVFQQEAHANLFNNGRH		*S. maltophilia*	
	NLDAKVFASQ NKLANGFEFQRN GAGLDYSHINGHGASLTHSNF			
	PGIGQQLGLDGRANLWSSPNRAT-TLDLTGSASKWTSGPFANQKPNF			
	GAGLGLSHHFG			
Attacin B from *Hyphantria cunea*	QAQGSLTFNGDGSAGVGAKVPLVGNDRNVLSAIGSVNLDDRMKLASKG	+5	*E. coli*	0.1 and 1.0 µg of protein (radial diffusion assay)
	IGLAFDNANGHGVSVMKET IPGFGSKLTGAGHATLFNNDNHNIGANA		*C. freundii*	
	FVSRNMPNIPNVPNFNTVGGG LDYTYNNKVGASLGMAHTPFLQRKD		*C. albicans*	
	YSAMGNLNVFRNPTSTVDFSAGFKKFDSPFPKSGWKPNLGLSFERIF			
Attacin from *Spodoptera exigua*	QAQGSVTLNSDGGMGLGAKI-PLANNDRNVLSAVGSMDLNNN-MNPTSKG	+4	*E. coli* DH5α	1, 0.5, 0.1, and 0.05 µg of protein (radial diffusion assay)
	FGLALDNVNGHGLTVMKES VPG-FGDRLSGAGKLNVFHNDNHNVAVTGSL		*P. cichorii*	
	TRNMPSIPNVPNFN-TIGGGVDYMYKNKVGASLGMASTPFLDRKDYSAMG		*B. subtilis*	
	NLNLFRSPTTSVDFSGGFKK-FESPFMSSGWKPNFGLTFGRSF		*L. monocytogenes*	
Attacin A1 from *Glossina morsitans morsitans*	GQFGGTVSSNPNGGLDVNARLSKTIGDPNANVVGGVFAAGNTDGGPATRGA	+4	*E. coli* K12	0.3 µM (minimum bactercidal concentration, MBC)
	FLAANKDGHGLSLQHSKTDNFGSSLTSSAHAHLFNDKTHKLDANAFH			
	SRTHLDNGFKFDRVGGGLRYDHVTGHGASLTASRIPQLDMNTLGLTGKANL		*Trypanosoma brucei*	0.075 µM (50 % minimal inhibitory concentration, MIC_50_)
	WSSPNRATTLDLTGGVSKHFGGPFDGQTNKQIGLGLNSRF			
Attacin from *Hermetia illucens*	KFLGNPNHNIGGGVFAAGN-TRSNTPSLGAF-GTLNLKDHSLGVSKTITPGVS	+10	*E. coli*	N.D.
	DTFSQNARLNILKTPDHR-VDANVFNSHTRLN-NGFAFDKRRGGSLDYTH		*S. aureus* KCCM 40881	
	RAGHGLSLGA-SHIP-KFGTTAELTGKANLWRSPSGLSTFDLTGSASRTF			
	GGPMAGRNNFGAGLGFSHRF			

N.D. = not determined. The results were descriptive in the considered papers and no numerical values could be obtained from the available data.
